# Cellular signatures underlying functional resilience in presymptomatic frontotemporal dementia

**DOI:** 10.1093/brain/awaf443

**Published:** 2025-11-24

**Authors:** Kamen A Tsvetanov, Maura Malpetti, P Simon Jones, Timothy Rittman, David J Whiteside, Alexander G Murley, Richard Bethlehem, Casey Paquola, Enrico Premi, Arabella Bouzigues, Lucy L Russell, Phoebe H Foster, Eve Ferry-Bolder, John C van Swieten, Lize C Jiskoot, Harro Seelaar, Raquel Sanchez-Valle, Robert Laforce, Caroline Graff, Daniela Galimberti, Rik Vandenberghe, Alexandre de Mendonça, Pietro Tiraboschi, Isabel Santana, Alexander Gerhard, Johannes Levin, Sandro Sorbi, Markus Otto, Maxime Bertoux, Thibaud Lebouvier, Simon Ducharme, Chris R Butler, Isabelle Le Ber, Elizabeth Finger, Maria Carmela Tartaglia, Mario Masellis, Matthis Synofzik, Fermin Moreno, Barbara Borroni, Jonathan D Rohrer, James B Rowe, the Genetic FTD Initiative, GENFI, Rhian Convery, Martina Bocchetta, David Cash, Sophie Goldsmith, Kiran Samra, David L Thomas, Thomas Cope, Maura Malpetti, Antonella Alberici, Enrico Premi, Roberto Gasparotti, Emanuele Buratti, Valentina Cantoni, Andrea Arighi, Chiara Fenoglio, Vittoria Borracci, Maria Serpente, Tiziana Carandini, Emanuela Rotondo, Giacomina Rossi, Giorgio Giaccone, Giuseppe Di Fede, Paola Caroppo, Sara Prioni, Veronica Redaelli, David Tang-Wai, Ekaterina Rogaeva, Johanna Krüger, Miguel Castelo-Branco, Morris Freedman, Ron Keren, Sandra Black, Sara Mitchell, Christen Shoesmith, Robart Bartha, Rosa Rademakers, Jackie Poos, Janne M Papma, Lucia Giannini, Liset de Boer, Julie de Houwer, Rick van Minkelen, Yolande Pijnenburg, Benedetta Nacmias, Camilla Ferrari, Cristina Polito, Gemma Lombardi, Valentina Bessi, Enrico Fainardi, Stefano Chiti, Mattias Nilsson, Henrik Viklund, Melissa Taheri Rydell, Vesna Jelic, Linn Öijerstedt, Tobias Langheinrich, Albert Lladó, Anna Antonell, Jaume Olives, Mircea Balasa, Nuria Bargalló, Sergi Borrego-Ecija, Ana Verdelho, Carolina Maruta, Tiago Costa-Coelho, Gabriel Miltenberger, Frederico Simões do Couto, Alazne Gabilondo, Ioana Croitoru, Mikel Tainta, Myriam Barandiaran, Patricia Alves, Benjamin Bender, David Mengel, Lisa Graf, Annick Vogels, Mathieu Vandenbulcke, Philip Van Damme, Rose Bruffaerts, Koen Poesen, Pedro Rosa-Neto, Maxime Montembault, Raphaella Lara Migliaccio, Ninon Burgos, Daisy Rinaldi, Catharina Prix, Elisabeth Wlasich, Olivia Wagemann, Sonja Schönecker, Alexander Maximilian Bernhardt, Anna Stockbauer, Jolina Lombardi, Sarah Anderl-Straub, Adeline Rollin, Gregory Kuchcinski, Vincent Deramecourt, João Durães, Marisa Lima, Maria João Leitão, Maria Rosario Almeida, Miguel Tábuas-Pereira, Sónia Afonso, João Lemos

**Affiliations:** Department of Clinical Neurosciences and Cambridge University Hospitals NHS Trust, University of Cambridge, Cambridge CB23 3EB, UK; Department of Psychology, University of Cambridge, Cambridge CB2 1NF, UK; Department of Clinical Neurosciences and Cambridge University Hospitals NHS Trust, University of Cambridge, Cambridge CB23 3EB, UK; UK Dementia Research Institute, University of Cambridge, Cambridge CB2 0AH, UK; Department of Clinical Neurosciences and Cambridge University Hospitals NHS Trust, University of Cambridge, Cambridge CB23 3EB, UK; Department of Clinical Neurosciences and Cambridge University Hospitals NHS Trust, University of Cambridge, Cambridge CB23 3EB, UK; Department of Clinical Neurosciences and Cambridge University Hospitals NHS Trust, University of Cambridge, Cambridge CB23 3EB, UK; Department of Clinical Neurosciences and Cambridge University Hospitals NHS Trust, University of Cambridge, Cambridge CB23 3EB, UK; Department of Psychiatry, University of Cambridge, Cambridge CB2 8AH, UK; Institute for Neuroscience and Medicine, INM-7, Forschungszentrum Jülich, Jülich 52428, Germany; Neurology, Department of Neurological and Vision Sciences, ASST Spedali Civili, Brescia 25123, Italy; Dementia Research Centre, Department of Neurodegenerative Disease, UCL Queen Square Institute of Neurology, London WC1E 6BT, UK; Dementia Research Centre, Department of Neurodegenerative Disease, UCL Queen Square Institute of Neurology, London WC1E 6BT, UK; Dementia Research Centre, Department of Neurodegenerative Disease, UCL Queen Square Institute of Neurology, London WC1E 6BT, UK; Dementia Research Centre, Department of Neurodegenerative Disease, UCL Queen Square Institute of Neurology, London WC1E 6BT, UK; Department of Neurology, Erasmus Medical Centre, Rotterdam 3015 GD, Netherlands; Department of Neurology, Erasmus Medical Centre, Rotterdam 3015 GD, Netherlands; Department of Neurology, Erasmus Medical Centre, Rotterdam 3015 GD, Netherlands; Alzheimer’s Disease and Other Cognitive Disorders Unit, Neurology Service, Hospital Clínic, Institut d’Investigacións Biomèdiques August Pi I Sunyer, University of Barcelona, Barcelona 08036, Spain; Clinique Interdisciplinaire de Mémoire, Département des Sciences Neurologiques, CHU de Québec, and Faculté de Médecine, Université Laval, Québec, QC G1V 4G2, Canada; Department of Neurobiology, Care Sciences and Society; Center for Alzheimer Research, Division of Neurogeriatrics, Bioclinicum, Karolinska Institutet, Solna 171 65, Sweden; Unit for Hereditary Dementias, Theme Inflammation and Aging, Karolinska University Hospital, Solna SE-17176, Sweden; Fondazione Ca’ Granda, IRCCS Ospedale Policlinico, Milan 20122, Italy; University of Milan, Centro Dino Ferrari, Milan 20122, Italy; Laboratory for Cognitive Neurology, Department of Neurosciences, KU Leuven, Leuven 3001, Belgium; Neurology Service, University Hospitals Leuven, Leuven 3000, Belgium; Leuven Brain Institute, KU Leuven, Leuven 3000, Belgium; Faculty of Medicine, University of Lisbon, Lisbon 1649-028, Portugal; Fondazione IRCCS Istituto Neurologico Carlo Besta, Milano 20133, Italy; University Hospital of Coimbra (HUC), Neurology Service, Faculty of Medicine, University of Coimbra, Coimbra 3004-531, Portugal; Center for Neuroscience and Cell Biology, Faculty of Medicine, University of Coimbra, Coimbra 3004-531, Portugal; Division of Psychology Communication and Human Neuroscience, Wolfson Molecular Imaging Centre, University of Manchester, Manchester M20 3LJ, UK; Department of Nuclear Medicine, Center for Translational Neuro- and Behavioral Sciences, University Medicine Essen, Essen 45147, Germany; Department of Geriatric Medicine, Klinikum Hochsauerland, Arnsberg 59755, Germany; Department of Neurology, Ludwig-Maximilians Universität München, Munich 80802, Germany; German Center for Neurodegenerative Diseases (DZNE), Munich 81377, Germany; Munich Cluster of Systems Neurology (SyNergy), Munich 81377, Germany; Department of Neurofarba, University of Florence, Florence 50139, Italy; IRCCS Fondazione Don Carlo Gnocchi, Florence 50139, Italy; Department of Neurology, University of Ulm, Ulm 89081, Germany; Lille Neuroscience & Cognition U1172, University of Lille, Inserm, CHU Lille, Lille 59000, France; Lille Neuroscience & Cognition U1172, University of Lille, Inserm, CHU Lille, Lille 59000, France; Douglas Mental Health University Institute, Department of Psychiatry, McGill University, Montreal, QC H4H 1R3, Canada; McConnell Brain Imaging Centre, Montreal Neurological Institute, McGill University, Montreal, QC H4H 1R3, Canada; Nuffield Department of Clinical Neurosciences, Medical Sciences Division, University of Oxford, Oxford OX3 7JX, UK; Department of Brain Sciences, Imperial College London, London SW7 2AZ, UK; Sorbonne Université, Paris Brain Institute – Institut du Cerveau – ICM, Inserm U1127, CNRS UMR 7225, AP-HP - Hôpital Pitié-Salpêtrière, Paris 75013, France; Centre de référence des démences rares ou précoces, IM2A, Département de Neurologie, AP-HP - Hôpital Pitié-Salpêtrière, Paris 75013, France; Département de Neurologie, AP-HP - Hôpital Pitié-Salpêtrière, Paris 75013, France; Department of Clinical Neurological Sciences, University of Western Ontario, London, ON, Canada N6A 3K7; Tanz Centre for Research in Neurodegenerative Diseases, University of Toronto, Toronto, ON, Canada M5T 0S8; Sunnybrook Health Sciences Centre, Sunnybrook Research Institute, University of Toronto, Toronto, ON, Canada M5T 2S8; Department of Neurodegenerative Diseases, Hertie-Institute for Clinical Brain Research and Center of Neurology, University of Tübingen, Tübingen 72076, Germany; Center for Neurodegenerative Diseases (DZNE), Tübingen 72076, Germany; Cognitive Disorders Unit, Department of Neurology, Hospital Universitario Donostia, San Sebastian 20014, Spain; Neurosciences Area, Group of Neurodegenerative Diseases, Biogipuzkoa Health Research Institute, San Sebastian 20014, Spain; Center for Biomedical Research in Neurodegenerative Disease (CIBERNED), Carlos III Health Institute, Madrid 28029, Spain; Department of Clinical and Experimental Sciences, University of Brescia, Brescia 15-25121, Italy; Molecular Markers Laboratory, IRCCS Istituto Centro San Giovanni di Dio Fatebenefratelli, Brescia 15-25121, Italy; Dementia Research Centre, Department of Neurodegenerative Disease, UCL Queen Square Institute of Neurology, London WC1E 6BT, UK; Department of Clinical Neurosciences and Cambridge University Hospitals NHS Trust, University of Cambridge, Cambridge CB23 3EB, UK; MRC Cognition and Brain Science Unit, Department of Psychiatry, University of Cambridge, Cambridge CB2 7EF, UK

**Keywords:** resilience, frontotemporal dementia (FTD), familial, presymptomatic, functional magnetic resonance imaging (fMRI), network connectivity

## Abstract

Frontotemporal dementia (FTD) shows autosomal dominant transmission in up to a third of families, enabling the study of presymptomatic and prodromal phases. Despite self-reported well-being and normal daily cognitive functioning, brain structural changes are evident a decade or more before the expected onset of disease. This divergence between cognitive function and brain structure contrasts with the coupling of structural and functional decline after symptom onset. In healthy ageing, it has been shown that functional connectivity is a better predictor of cognitive function than volumetric structural imaging. We previously proposed that in the presymptomatic phase of genetic FTD, the maintenance of brain functional network integrity enables carriers of pathogenic variants to sustain cognitive performance. However, prior work has focused on a small number of, often predefined, networks. This provides a limited and potentially biased characterization of the substrates and moderators of brain network integration.

Here, we test the hypothesis that brain-wide functional integration in FTD determines resilience to progressive pathology before symptom onset. We assess functional connectome integration in 289 presymptomatic carriers of pathogenic variants associated with FTD using functional MRI in relation to cognition and contrast with 271 family members without pathogenic variants. Because structural atrophy, functional integration and cognitive profiles are multivariate, we used canonical correlation models, supplemented by multiple linear regression models for each imaging modality.

We confirmed progressive atrophy and normal cognitive function in presymptomatic carriers compared to non-carriers. Notably, functional integration was preserved in presymptomatic carriers across age, while it declined in familial non-carriers. The strongest effects were observed in cognitive control networks. The changes in functional integration in presymptomatic carriers were behaviourally relevant and independent of the severity of atrophy, suggesting a resilience mechanism in those at risk of dementia. To generate hypotheses about the genetic and neurometabolic basis of resilience, we assessed the spatial overlap between behaviourally-relevant functional integration maps and gene transcription profiles. These spatial correlations suggested resilience signatures to glial cell composition (astrocytes, microglia, oligodendrocytes), revealing cellular mechanisms inaccessible to standard neuroimaging.

Our findings suggest that resilience to atrophy is associated with enhanced functional integration, protecting against clinical conversion for many years in individuals at risk of dementia. This result has implications for the design of presymptomatic disease-modifying therapy trials and gives hope for therapeutic strategies aimed at enhancing resilience and ability to maintain function despite the presence of genetically determined neuropathology.

## Teaser

Tsvetanov *et al.* report that presymptomatic carriers of FTD-associated variants are able to maintain cognitive function despite brain atrophy owing to preservation of network connectivity that would normally decline with ageing. This ‘functional resilience’ correlates with glial cell distribution, suggesting a protective role for these cells.

## Introduction

Neurodegeneration begins many years before the onset of symptoms in people at risk of dementia. Neuropathological and structural changes during this presymptomatic period are well established in Alzheimer’s disease, Huntington’s disease and frontotemporal dementia (FTD), using structural brain imaging, radiotracer imaging and fluid biomarkers. However, it remains unclear why people remain functionally resilient to advancing neuropathology. We propose that the integration of functional network organization during these presymptomatic stages confers resilience to the effects of neurodegeneration, i.e. enabling cognitive function despite atrophy. However, identifying the mechanisms underlying this resilience requires methodological advances. This study introduces two key innovations: (i) a brain-wide, data-driven approach that overcomes regional bias limitations of previous network-focused studies; and (ii) a framework linking functional resilience patterns to cellular transcriptomic profiles. Together, these advances provide the means to identify where resilience operates across the brain and the cellular signatures that moderate the effects of neurodegeneration.

Maintaining cognitive abilities depends on the coupling of structural and functional properties of brain networks as people age.^1-3^ This phenomenon is amplified in those at risk of dementia.^4,5^ The links between structure, function and performance have been influential in developing current models of neurocognitive ageing and dementia.^6-9^ These associations contrast with the emerging evidence of neuropathological and structural changes many years before the onset of symptoms of Alzheimer’s disease, Huntington’s disease and FTD.^10-15^ This begs the question, why are some people resilient to pathology for so long?

Genetic FTD provides a model to study resilience. FTD has highly-penetrant gene variants. This allows one to study how the brain maintains good cognitive function during the presymptomatic disease stages, despite ongoing neurodegeneration. Pathogenic variants in three main genes cause 10%–20% of FTD cases: chromosome 9 open reading frame 72 (*C9orf72*), granulin (*GRN*) and microtubule-associated protein tau (*MAPT*). Symptom timing varies between genes^16,17^ and even within the same gene variant.^18^ Environmental and secondary genetic factors can influence this.^19,20^ However, all three pathogenic variants cause significant structural brain changes in key regions. These changes happen over 10 or more years before the symptoms start,^14,21^ as confirmed in longitudinal studies.^22-24^

We recently proposed that the maintenance of functional network integrity helps presymptomatic carriers with pathogenic variants to stay well despite progressive atrophy.^4,5^ This connectivity-cognition relationship was stronger in older adults^25^ and individuals with high dementia risk.^26,27^ This finding generalized across different brain imaging methods,^28-30^ cognitive states^31,32^ and analytical approaches.^2,33,34^ However, previous studies looked at only a few brain networks (e.g. salience network, default mode network or frontoparietal network), providing a limited and potentially biased characterization of functional integration substrates. Here we introduce a brain-wide, data-driven approach, extending the network-based approach in Bethlehem *et al.*^33^ This captures the distributed nature of cognitive substrates and neurodegenerative disease targets, which extend across multi-level, interactive networks, not just single brain regions or networks.^35,36^ These hierarchical patterns could be particularly relevant given their differential vulnerability in FTD.^37,38^

Functional MRI (fMRI) has been the primary tool for investigating functional networks in dementia, but it has several limitations as a means to understand the mechanisms of resilience.^39,40^ Data-driven, brain-wide approaches have been developed to link functional measures to behaviour.^2,6,26,34,41-45^ However, connectome-based methods remain difficult to interpret^2,46^ and lack direct mappings between biological mechanisms and functional connectivity observations. Recent imaging transcriptomics advances enabled the study of transcriptional correlates of neural phenotypes.^47,48^ This offered a new means for evaluating pathophysiological mechanisms on a cell-systems level.^49^ Here, we propose an extension of this method that identifies behaviourally-relevant, brain-wide patterns of functional integration, linked to gene transcription profiles.^47,50^ This approach overcomes traditional neuroimaging challenges and reveals how functional resilience relates to cellular and molecular variations. This allows us to uncover insights typically inaccessible to standard neuroimaging techniques.

We sought to identify the brain-wide topology of network integration in presymptomatic FTD. This addresses the challenge that cognition and neurodegeneration are distributed across multi-level networks, not single regions. We used a multivariate approach to identify differences in brain-behaviour relationships between presymptomatic carriers of pathogenic variants in genes associated with FTD and non-carriers from the same families. We employed and extended multi-dimensional brain network analysis^33^ across cortical organization levels,^35,36^ which are known to be differentially affected in FTD.^37,38^ Our hypothesis was that higher-order cognitive networks confer cognitive reliance in at-risk individuals. They would preserve function despite structural degradation. We explored how network integration patterns correspond to regional cell-type distributions. We propose that functional network resilience reflects cell-type decomposition essential for neuronal homeostasis under stress.

## Materials and methods

### Participants

**Figure 1:**
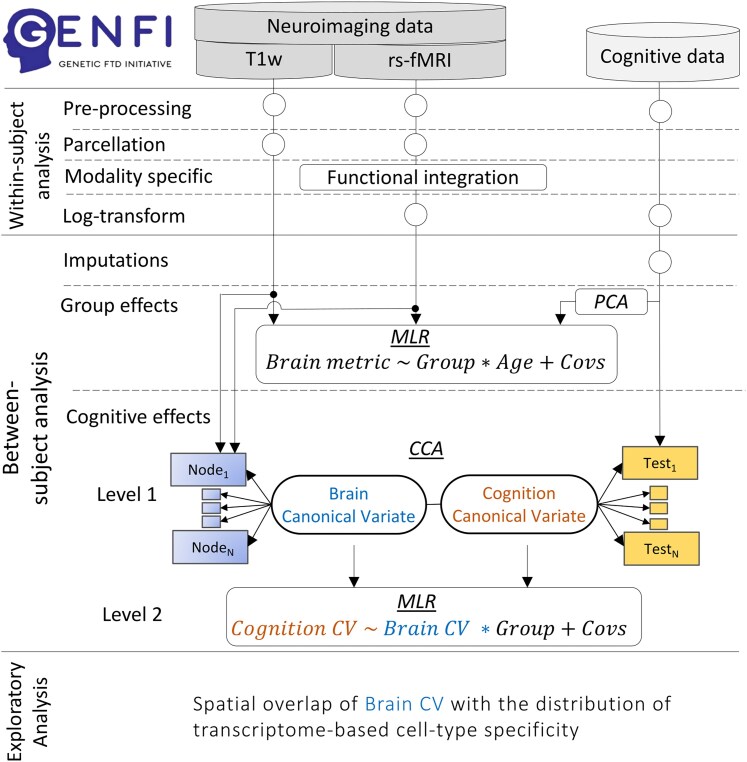
**Schematic representation of modality datasets and their processing pipelines at the within-subject level and data-reduction techniques and analytical strategy at the between-subject level to test for group and cognitive effects of brain metrics**. CCA = canonical correlation analysis; Covs = covariates of no interest; CVs = canonical variates from CCA analysis; Group = genetic status, i.e. presymptomatic carrier versus non-carrier; MLR = multiple linear regression; rs-fMRI = resting state functional MRI; T1w = T1-weighted image acquisition.

**Figure 2:**
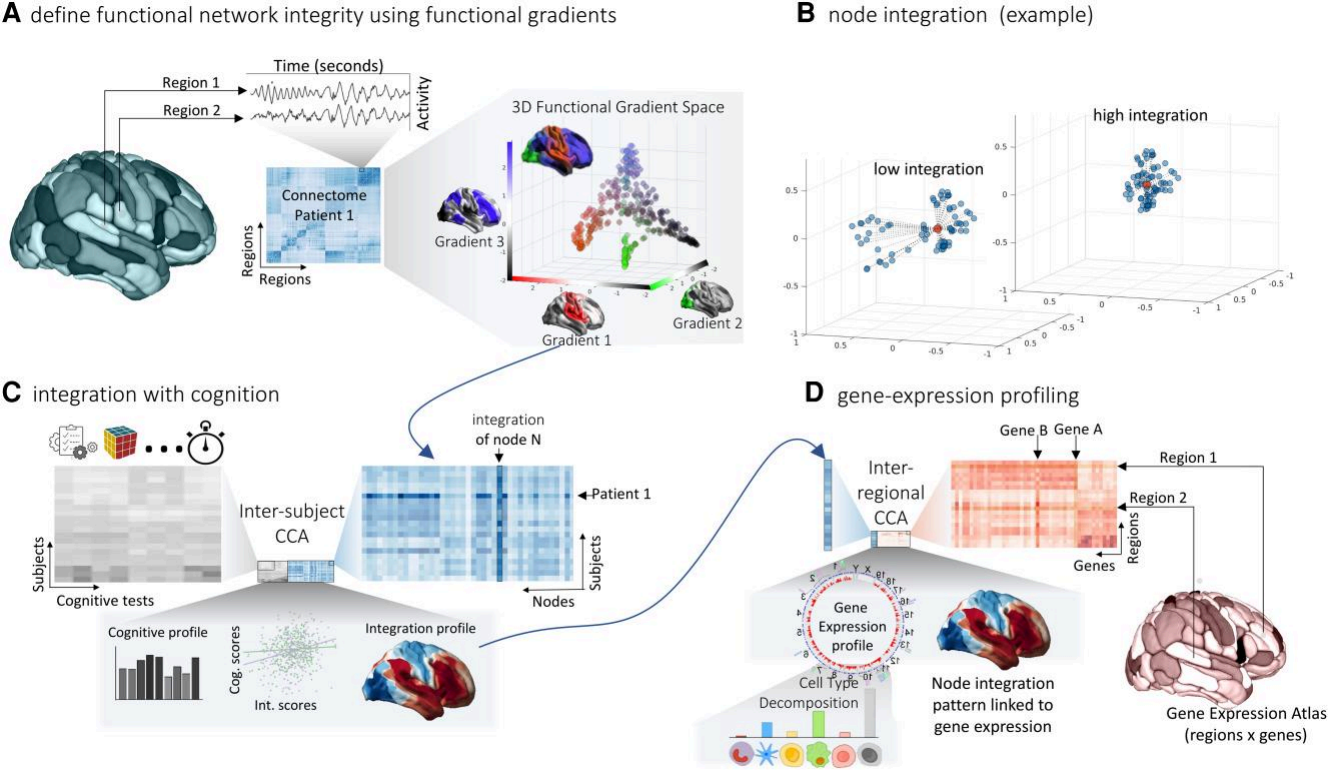
**Schematic representation of defining network integration and its integration with cognition and gene-expression profiling.** (**A**) Functional network integration using functional gradient extraction: Resting-state functional MRI data were processed and parcellated into Glasser’s 360 nodes to estimate functional connectomes in terms of node-to-node functional connectivity. Individual estimates of the observed functional connectomes were concatenated as inputs to the diffusion embedding approach. The first three gradients are projected into 3D gradient space and shaded according to the cortical surface colour map above the scatter plot. Each filled circle indicates the location of a node in this 3D space, with each functional gradient projected onto the cortical surface next to the corresponding axis. Gradient 1 separates somatomotor and auditory cortex (red) from default mode regions (black). Gradient 2 extends between visual cortex (green) and default mode regions (black). Gradient 3 separates high-order regions (blue) from default mode and sensorimotor regions (black, red, green). The gradient space was constructed in a hold-out sample. (**B**) Schematic illustration of the proposed integration metric for a simulated node (red) and its community of nodes (blue) with low and high integration (*left* and *right*, respectively). Node integration was estimated as the inverse sum squared of Euclidean distances from each community node (blue) to the red node. Lower values represent a wider distribution of the community in the 3D gradient space, i.e. a more dispersed community (*left*). Higher values represent a denser distribution of the community, i.e. an integrated community (*right*). The approach was repeated for each Glasser node in **A** for each individual’s own gradient space, where the node community was based on the top 10% closest nodes in the hold-out template. (**C**) Link between network integration and cognitive function: individual estimates of functional integration and performance across a wide range of cognitive tests were concatenated (subjects by integration across regions and subjects by performance across cognitive tests) as inputs to inter-subject canonical correlation analysis (CCA). CCA identified sources of signal in network integration and cognition that were related across individuals in terms of functional integration patterns, cognitive profiles and subjects’ scores. The second-level analysis tests whether the relationship between integration and cognition subject scores was stronger for presymptomatic carriers (evidence for functional resilience). (**D**) Gene-expression profiling of functional integration: the spatial correspondence between the behaviour-relevant network integration map and gene expression data was integrated using inter-regional CCA. The gene expression profile expressed highly by the functional integration map was analysed using cell-type decomposition to identify cell-type enrichment based on the extent to which genes were expressed in the transcriptome map.

The study design and the principal data processing pipelines are summarized in Figs 1 and 2. Twenty-five research sites across Europe and Canada recruited participants as part of an international multicentre partnership, the Genetic Frontotemporal Initiative (GENFI).^14,21^ The study was given a favourable opinion by the Cambridge 2 Research Ethics Committee REC 17/EE/0032 IRAS ID 204052. Informed consent was obtained by all human participants or, when not possible, assent was obtained with proxy consent. Inclusion criteria included anyone over the age of 18, who is symptomatic or an asymptomatic first-degree relative.

Human participants were genotyped and grouped according to whether they carried a pathogenic variant in *MAPT* or *GRN* genes or an expansion in C*9or*f72. Carriers were classified as either symptomatic or presymptomatic based on clinical evaluation. Participants were classified as symptomatic if the clinician judged that symptoms were present, progressive and consistent with a diagnosis of a neurodegenerative disorder. A comparison group acted as controls, termed non-carriers, and comprised mutation-negative family members. In this study, based on data freeze 5, we focused on non-carriers (NC, *n* = 271) and presymptomatic carriers (PSC, *n* = 289) with usable structural and resting-state fMRI data. Participants and site investigators were blinded to the research genotyping, although a minority of participants had undergone predictive genetic testing outside the GENFI study. See Table 1 for demographic, behavioural, cognitive and neuropsychological information about both groups.

**Table 1:** Demographics and cognitive test measures with statistical tests assessing differences between non-carrier and presymptomatic carrier groups

	Gene status group		Statistical tests^a^	
	NC	PSC	χ^2^ or *F*-test	*P*-value
*N*	271	289	–	*–*
**Mutated gene, *n* (%)**	–	–	–	–
*MAPT*	–	54 (18.7)	–	*–*
*GRN*	–	126 (43.6)	–	*–*
*C9orf72*	–	107 (37)	–	*–*
*TBK1*	–	2 (0.7)	–	*–*
**Gender, *n* (%)**	–	–	0.02	0.896
Male	114 (42.1)	120 (41.5)	–	–
**Handedness, *n* (%)**	–	–	1.2	0.274
Right-handed	248 (91.5)	257 (88.9)	–	–
**Age, years**	–	–	2.31	0.13
Mean/SD	46/13	44/11	–	–
Range, min/max	19/86	20/75	–	–
**Education, years**	–	–	0.18	0.674
Mean/SD	15/3	15/3	–	–
Range, min/max	5/24	5/24	–	–
**Cognitive test**				
Digit Span—Forwards	7.7 (2)	8 (2.1)	3.14	0.076
Digit Span—Backwards	6.7 (2.2)	6.9 (2.2)	0.89	0.372
Digit Symbol Task	58 (14.3)	57.5 (13.4)	0.19	0.661
Trail Making Test Part A	27.7 (14.4)	27.8 (10.6)	1.71	0.192
Trail Making Test Part B	66 (33.2)	67.8 (40.2)	0.06	0.800
Verbal Fluency—Letter	41.2 (12.7)	40 (14.3)	1.23	0.268
Verbal Fluency—Animal	23.4 (5.8)	24.1 (5.8)	3.12	0.078
Boston Naming Test	31.8 (6.5)	30.8 (6.8)	0.15	0.697
Block Design	48 (15.1)	47.6 (15.8)	0.01	0.975
FCSRT D Free	12.2 (2.5)	11.7 (2.9)	2.44	0.118
FCSRT Free	31.8 (6.5)	30.8 (6.8)	1.75	0.186
Stroop Colour	28.6 (5.8)	29.7 (7.4)	2.07	0.151
Stroop Ink	50.4 (12.6)	52.6 (16.3)	1.08	0.299
Stroop Word	22.5 (5.4)	22.5 (5.6)	0.01	0.983

Demographics and cognitive test measures are presented as means and standard deviations (SD). FCSRT = Free and Cued Selective Reminding Test; NC = non-carrier; PSC = presymptomatic carrier.

^a^Statistical test to indicate whether demographics vary between NC and PSC groups.

### Neurocognitive assessment

Each participant completed a standard clinical assessment consisting of medical history, family history, functional status and physical examination, in complement with collateral history from a family member or a close friend. In the current study, 14 behavioural measures of cognitive function were correlated with neuroimaging measures. These included the Uniform Data Set^51^: Digit Span forwards and backwards from the Wechsler Memory Scale-Revised, a Digit Symbol Task, Parts A and B of the Trail Making Test, the short version of the Boston Naming Test, and Category Fluency (animals). Additional tests included Letter Fluency and Wechsler Abbreviated Scale of Intelligence Block Design task, Free and Cued Selective Reminding Test (FCSRT) and Stroop tests. Latency measures for the Trail Making Test and Stroop tests were log-transformed and inverted so that higher values across all tests reflect better performance. Rate of missing neurocognitive data varied between 0% and 10%; hence, to increase statistical power and efficiency, missing data were imputed before further statistical analyses. Incomplete variables were imputed under fully conditional specification, using the default settings of the multivariate imputation by chained equations (MICE) in R.^52^

To test for differences in performance across cognitive measures, we performed principal component analysis as a widely used dimensionality reducing approach.^53-55^ For this purpose, we used MATLAB’s function *pca.m* with default settings, where the number of components was determined using Horn’s parallel analysis^56,57^ using 10 000 permutations. All variables were normalized to a mean of 0 and standard deviation of 1 prior to principal component analysis.

### Neuroimaging assessment

#### T1-weighted image acquisition and processing

Figures 1 and 2 provide a schematic representation of the imaging data processing pipeline and the analysis strategy used to link brain and behaviour data. MRI data were acquired using 3 T with optimized scanning protocols to maximize synchronization across scanners and sites.^14,21^ A 3D structural MRI was acquired on each participant using T1-weighted magnetization prepared rapid gradient echo (MPRAGE) sequence over at least 283 s (283–462 s) and had a median isotropic resolution of 1.1 mm, repetition time of 2000 ms (6.6–2400), echo time of 2.9 ms (2.8–4.6 ms), inversion time of 8 ms (8–9 ms), and field of view 256 × 256 × 208 mm. The imaging data were analysed using FSL pipelines^58,59^ and modules which called relevant functions from SPM12.^60^ Native-space segmentation of grey matter, white matter and CSF tissue and warps for normalization to the Montreal Neurological Institute template space were estimated using FSL. In addition, anatomical images were processed and individually evaluated in FreeSurfer^61^ data were quality-control checked by semiautomated scripts monitored as previously described.^62^

#### Functional MRI image acquisition and processing

##### Preprocessing

For resting-state fMRI measurements, echo-planar imaging (EPI) data were acquired with at least 6 min of scanning. Analogous imaging sequences were developed by the GENFI Imaging Core team and used at each GENFI study site to accommodate different scanner models and field strengths. EPI data were acquired over at least 308 s (median 500 s) and had a median repetition time of 2500 ms (2200–2500 ms), echo time of 30 ms, flip angle of 80 ms (80–85 ms), in-plane resolution of 2.72 × 2.72 mm (2.72–3.50 × 2.72–3.25) and slice thickness of 3.5 mm (2.72–3.5).

To ensure that potential group bias in head motion did not affect later analysis of connectivity, we took three further steps: (i) fMRI data were further post-processed using whole-brain independent component analysis (ICA) of single-subject time-series denoising, with noise components selected and removed automatically using *a priori* heuristics using the ICA-based algorithm^63^; (ii) post-processing of node-based time-series (see later); and (iii) a subject-specific estimate of head movement for each participant^64^ included as a covariate in group-level analysis.^34^

##### Post-processing and connectome estimation

For every individual, we projected ICA-denoised fMRI data to surface space and averaged vertex-based time series within 360 nodes from a multimodal parcellation of the human cerebral cortex.^65^ We post-processed nodal time-series to further reduce the effects of noise confounds on functional connectivity effects using the general linear model (GLM).^34^ This model included linear trends, expansions of realignment parameters, as well as average signal in white matter and CSF, including their derivative and quadratic regressors^66^ and CompCorr regressors^67^ from the time courses of each node. The average white matter and CSF signals and CompCor signals were created by using the signal across all voxels with associated tissue type as defined by FSL’s segmentation. A band-pass filter (0.0078 to 0.1 Hz) was implemented by including a discrete cosine transform set in the GLM, ensuring the nuisance regression and filtering were performed simultaneously.^68-70^ Finally, the functional connectivity between each pair of nodes was computed using Pearson correlation on postprocessed time series. Individual connectivity matrices were subjected to row-wise thresholding (top 10% of edges maintained) and converted into normalized angle matrices.^33,35,71^

##### Gradient extraction

To determine the gradients of subject-level connectomes, we employed diffusion map embedding as a non-linear dimensionality reduction technique,^72^ following the methodology described in Bethlehem *et al.*^33^ This approach builds upon the foundational work of Margulies *et al.*,^35^ who showed that functional gradient frameworks capture brain-wide organizational patterns of brain networks across cortical hierarchies.^36,73^ These hierarchical patterns are particularly relevant given their differential vulnerability in FTD.^37,38^ Since its introduction, the functional gradients framework has gained a widespread adoption and demonstrated high reproducibility^74^ that generalizes across species,^75,76^ neuroimaging modalities^77,78^ and brain disorders^38,79-82^ Importantly, the method offered superior robustness to confounds and enhanced sensitivity to cognitive decline relative to conventional connectivity analyses.^33^

First, a group-average functional connectivity matrix was constructed, thresholded, normalized and subjected to diffusion map embedding from an independent healthy adult cohort part of the Cambridge Centre for Ageing and Neuroscience, Cam-CAN, project (*n* = 637).^83,84^ The first three gradients explained 41.3% of the variance and were selected for further analysis. The gradients showed a functional differentiation running from sensory-to-transmodal abstract (G1), visual-to-transmodal abstract (G2) and somatomotor-to-transmodal executive (G3). This was in line with converging evidence that the transmodal cortex can be delineated into two sets of regions^85-87^: (i) regions hypothesized to serve more abstract functions, e.g. default mode network^88,89^; and (ii) regions hypothesized to serve intelligence and executive control.^44,90-92^ Together, these gradients described functional discrimination between sensory modalities and across levels of the cortical hierarchy (i.e. sensory processing, abstract thinking and higher-order cognition), which are known to be differentially affected in FTD.^37,38^ Regional gradient values reflected the expression of connectivity profiles in relation to that axis (e.g. two regions with similar gradient values exhibit a similar distribution of resting state functional connectivity along the sensory-abstract transmodal axis).

The gradient template generated in the CamCAN cohort was used as a reference to which each GENFI individual gradient map was aligned using Procrustes transformation.^93^ The degree of deformation and translation of the transformation matrix for an individual’s gradient to align to the gradient template was used to evaluate potential bias in the alignment procedure.

##### Node integration using multi-dimensional gradients

To investigate differences in multi-dimensional cortical organization, we quantified a new metric, termed here ‘node integration’. This metric aimed to provide a more granular representation of the previously proposed metric of network integration in this multi-dimensional functional connectivity space^33^ across seven large-scale networks.^94^ This allowed us to estimate a measure of integration at the regional level, rather than integration measures at the network level. Here, we focus on the first three gradients, forming a 3D space in which each axis reflects the values along each gradient. We calculated ‘node integration’ as the inverse of the sum squared of Euclidean distances from each community’s centre of gravity to each node in that community (i.e. moving from large-scale networks in Bethlehem *et al.*^33^ to communities, allowing us to focus on node integration within segregated subsystems of the brain). The node community was based on the top 10% closest nodes in the group average such that high values indicate high integration (Fig. 2B, top scatter plot) and low values indicate low integration, i.e. high dispersion (Fig. 2B, bottom scatter plot). The approach was repeated for each node in each participant’s own gradient space. For completeness, we also explored the consistency of effects across varying the thresholds (e.g. 5% versus 10% versus 15% versus 20%).

### Statistical analysis

Statistical analysis used MATLAB 2020b calling the packages as described below. We performed a descriptive analysis of all the characteristics from demographic, clinical and cognitive characteristics within the sample before integrating these with neuroimaging data as described below.

#### Group and progression effects in brain structure and function

To determine gene-carrier group effects and pre-disease progression effects on each brain metric (cortical thickness and functional integration) of each node we used a robust linear regression as implemented in MATLAB’s function *fitlm.m*. Gene-carrier group effects were modelled based on group identity (presymptomatic carrier versus non-carrier) based on whether an individual was a carrier of a pathogenic variant in *C9orf72*, *MAPT* or *GRN*. Pre-disease progression effects were modelled using the interaction between group identity and individual’s age, on the basis that the pre-disease progression process in presymptomatic carriers differs from the normal ageing process in non-carriers. GENFI’s large-sampled cohort was created using harmonized multi-site neuroimaging data. Although, scanning protocols were optimized to maximize comparability across scanners and sites,^14,21^ different scanning platforms can still introduce systematic differences that might confound true effects of interest.^95^ Therefore, in the analysis of neuroimaging data we included scanner site in addition to sex and head motion^96^ as additional covariates of no interest. The regression model was specified by Wilkinson’s notation,^97^ ‘Brain metric ∼ Group × Age + Sex + Head motion + Scanner site’ (where group refers to gene status: pathogenic variant carrier versus non-carrier) and fitted for each node separately. We tested the same model with cognitive function as a dependent variable. To test whether the effects of functional integration were independent of atrophy effects, an additional model was performed, including cortical thickness of the corresponding node as a covariate of no interest (i.e. ‘Functional integration ∼ Group × Age + Sex + Head motion + Scanner site + Cortical thickness’). Models were evaluated using 10 000 permutation tests and reported at *P*-perm < 0.05. A sensitivity analysis was performed using a multivariate approach, which is described in the next section.

#### Brain-behaviour relationships

For brain-behaviour analysis, we adopted a two-level analytical procedure.^5,25,27,32,98^ In the first-level analysis, we assessed the multidimensional brain-behaviour relationships using regularized canonical correlation analysis with permutation-based 10-fold cross-validation. This analysis described the linear relationships between the two multivariate sets of variables, namely node integration and behavioural performance, by providing pairs of canonical variates (CVs) (Node Integration-CVs and Cognition-CVs) as linear combinations of the original variables, which were optimized to maximize their correlation. Namely, Dataset 1 consisted of node integration across 360 nodes (i.e. Node integration dataset). Dataset 2 included the performance measures on the nine tests (i.e. Cognition dataset). Next, we tested whether the identified behaviourally-relevant CVs of node- integration were differentially expressed by non-carriers and presymptomatic carriers as a function of age. To this end, we performed a second-level analysis using robust linear regression. Independent variables included subjects’ node integration scores from first level canonical correlation analysis (CCA), group identity and their interaction term (e.g. Brain scores × Group). The dependent variable was subjects’ cognitive scores from the first level CCA (Cognition-CV). Given that the interaction effects were derived from continuous variables, we tested and interpreted interactions based on simple slope analysis and slope difference tests.^99-101^ Covariates of no interest included age, sex, head motion and site scanner.

#### Spatial covariance of behaviour-relevant node integration with transcriptome-based cell-type decompositions

To generate hypotheses about the genetic and neurometabolic basis of resilience, we assessed the spatial overlap between behaviourally-relevant functional integration map (Function-CV regional loadings; hereafter referred to as the ‘phenotypic map’) and cell-type parameters using gene transcription profiling maps.^102,103^ Spatial covariance between phenotypic map and gene expression across 9394 genes expressed in the human brain^47,48,50^ was based on CCA association using 10 000 spin-permutation-based 10-fold cross-validations.^5,98^ The spin-based permutations (p-spin) aimed to preserve spatial autocorrelation.^104-106^ The latent variable represented a spatial pattern of gene expression that correlated significantly with the phenotypic map associated with cognitive function. Full details of the processed transcriptomic data are available elsewhere.^47^ To identify the cellular origins of genes associated with the phenotypic map, we performed cell-type decomposition analysis following an established framework, as follows.^102,103^ Rank-ordered genes exhibiting high loadings (i.e. high correlations) with the phenotypic map (i.e. phenotype-relevant genes) were evaluated against cell-type-specific profiles from molecular human brain atlas.^107^ These cell-type-specific reference profiles represented gene expression signatures that characterize distinct cellular populations, derived from single-cell RNA sequencing where genes were classed by their preferential expression in specific cell types. A complete list of gene-to-cell assignments is available in Yang *et al.*^107^ This enabled us to test whether the phenotype-relevant genes were preferentially expressed in specific cell types across eleven major canonical cortical cell classes: astrocytes (Astro); brain endothelial cells (BEC); ependymal cells (Epend); macrophage/microglia (MacMic); meningeal fibroblasts (MFibro); neurons (Neuro); oligodendrocyte precursors (Opc); oligodendrocytes (Oligo); pericytes (Peri); perivascular fibroblasts (PFibro); and smooth muscle cells (SMC). To this end, we calculated the ratio of genes in each set preferentially expressed by each cell type (e.g. ratio for pericytes is calculated from the number of genes preferentially expressed in pericytes divided by the total number of genes). Gene sets were thresholded to include the top *n*% of genes with significant loadings, where the significance threshold varied from 0.001 to 1 (all genes). Statistical significance was determined using a null distribution of ratios based on 10 000 sets of random genes.^102^

## Results

### Participants

Characteristics of the presymptomatic carriers and non-carriers in the current GENFI sample are detailed in Table 1. In a group of 289 presymptomatic carriers, 18.7% carried a pathogenic variant in *MAPT*, 43.6% in *GRN*, 37% in *C9Orf72* and 0.7% in *TBK1*. Among the presymptomatic carriers, nearly 89% of presymptomatic carriers were right-handed, 42% were male, the average age was 44 years, and the average number of years spent in education was 15 years. Presymptomatic carriers did not differ from non-carriers across these demographic variables and individual cognitive tests (Table 1). The percentage of missing values across cognitive tests varied between 0% and 10%. Of 560 participants, 12 records were incomplete for Trail Making datasets; two, three and 14 records were incomplete for Digit Symbol, Boston Naming and Block Design datasets, respectively; four and six records were incomplete for Fluency Animals and Fluency Letter data; and 56 and 49 records were incomplete for FCSRT and Stroop data, respectively.

### Group differences

#### Age-related cognitive decline similar in both groups

**Figure 3:**
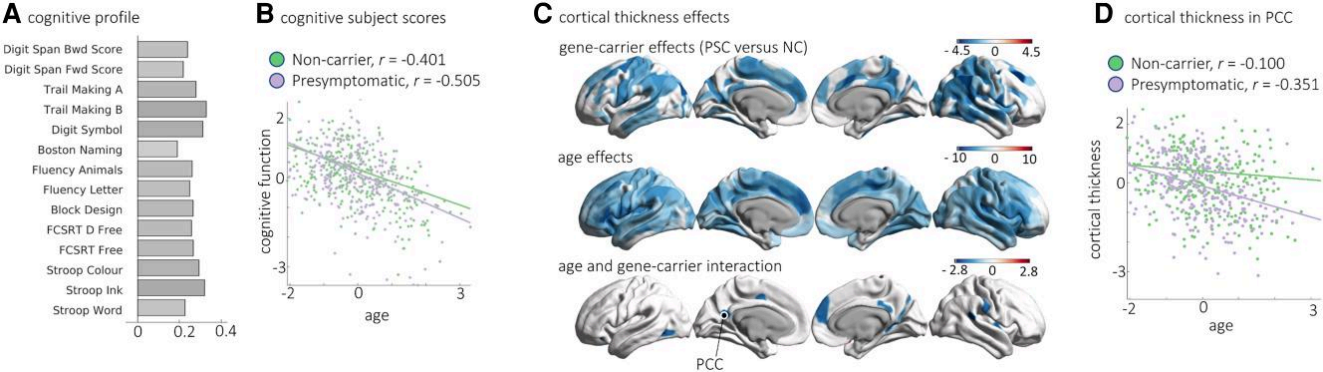
**Cognitive function and cortical thickness effects.** (**A**) Cognitive profile for the first and only significant principal component across cognitive measures. (**B**) Scatter plot of cognitive subject scores, visualizing the relationship between age and cognitive performance in each genetic status group separately. Each point indicates the strength with which an individual expresses the cognitive profile. (**C**) *Top*: Spatial visualization of cortical thickness effects for group differences between presymptomatic carriers (PSC) and non-carriers (NC), where the cooler colour (blue) scheme indicates the strength of the effect size of PSC, showing less cortical thickness than NC, while the warmer shades (red) indicate the opposite effect (i.e. PSC > NC). *Middle*: Age-related effects of cortical thickness. *Bottom*: Age and Group interaction effects. The cooler colour (blue) scheme indicates a disproportionately stronger age effect in PSC than in NC (i.e. disease progression). (**D**) Scatter plot of region-specific effects as indicated by the black-filled circle in **C** (*bottom*), visualizing the relationship between age and cortical thickness in each genetic status group separately. The *x*-axis range is −2 to 3, in increments of 1; the *y*-axis range is −3 to 2, in increments of 1. FCSRT = Free and Cued Selective Reminding Test; PCC = posterior cortical cortex.

Cognitive function was represented by a single principal component, *P* < 0.001, explaining 39.5% of the total variance across cognitive measures and reflecting overall cognitive function (Fig. 3A). The multiple linear regression model testing for overall group differences and group-age interaction in the cognitive function component between presymptomatic and non-carriers were not significant (*r* = −0.07, *P* = 0.084 and *r* = −0.05, *P* = 0.175). This indicates that cognitive function levels in presymptomatic carries were comparable to non-carriers, as expected (Fig. 3B).

#### Stronger cortical thinning with age in gene carriers

The multiple linear regression model testing for overall group differences in cortical thickness between presymptomatic and non-carriers was significant in frontal, temporal, parietal and subcortical regions (Fig. 3, top row), reflecting expected presymptomatic differences in brain-wide atrophy. There was also a strong brain-wide age-related difference in cortical thickness (Fig. 3, middle row). A small set of regions, including anterior and posterior cingulate and superior temporal gyrus, showed a significant interaction effect of age and gene status (Age:Gene Status; Fig. 3C, bottom row). The interaction effect reflected a stronger age-related decrease in cortical thickness for presymptomatic carriers versus non-carriers (i.e. disease progression of atrophy effects in presymptomatic FTD; Fig. 3D). Overall, the observed univariate effects were consistent with the multivariate analysis using canonical correlation analysis (Supplementary Fig. 3).

#### Functional integration declines with age in non-carriers, but is abnormally maintained in carriers of pathogenic variants

**Figure 4:**
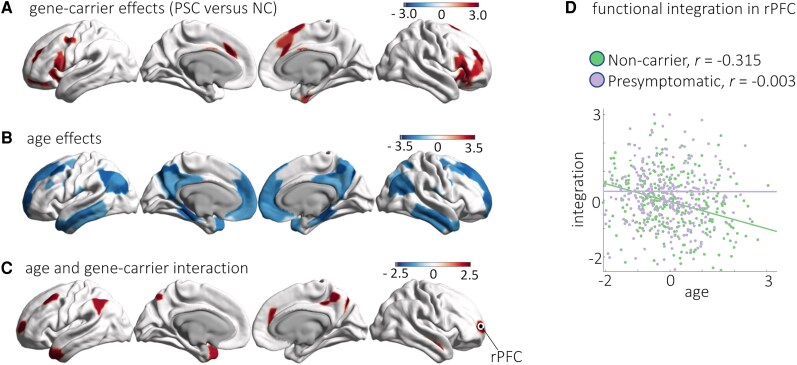
**Functional integration effects.** (**A**) Group differences between presymptomatic carriers (PSC) and non-carriers (NC), where warmer (red) shades indicate the strength of the effect size of PSC, showing higher functional integration than NC, while cooler (blue) shades indicate the opposite effect (i.e. PSC < NC). (**B**) Age-related effects of functional integration, indicated by cooler shades (blue), show an age-related decrease in functional integration. (**C**) Age and group interactions, indicated by warmer shades (red), show a disproportionately stronger age effect in NC versus PSC (i.e. functional resilience in PSC). Maps are thresholded at *P*-perm < 0.05, based on 10 000 permutations. For a more complete description of the spatial representations, see un-thresholded maps in Supplementary Fig. 1B. (**D**) Scatter plot of region-specific effects as indicated by a black filled-circle in **C**, visualizing the relationship between age and functional integration in each genetic status group, separately. *x*-axis range is −2 to 3, in increments of 1. rPFC = rostral prefrontal cortex.

The multiple linear regression test for group differences in functional integration was significant in several areas across the frontal cortex, including inferior frontal gyrus and anterior cingulate (Fig. 4A). In addition, we confirmed a strong age-related decrease in functional integration (Fig. 4B). There was a significant interaction between age and gene status (Age:Gene Status) in regions classically associated with cognitive control (anterior cingulate, dorsolateral prefrontal cortex and intraparietal sulcus), and the default mode (ventromedial cingulate, posterior cingulate and lateral temporal pole; Fig. 4C). The interaction effects were driven by a strong age-related decrease in functional integration among non-carriers, while presymptomatic carriers showed minimal to no age effects (Fig. 4D). This suggests that presymptomatic carriers sustained functional integration more strongly than their non-carrier peers in the approach to symptom onset. Sensitivity analyses confirmed that these effects were robust to varying levels of functional community thresholding (Supplementary Fig. 1), atrophy levels (Supplementary Fig. 2), and multivariate analysis (Supplementary Fig. 3).

### Brain-behaviour relationships

#### Cortical thickness-cognition relationships driven by age, not FTD gene status

**Figure 5:**
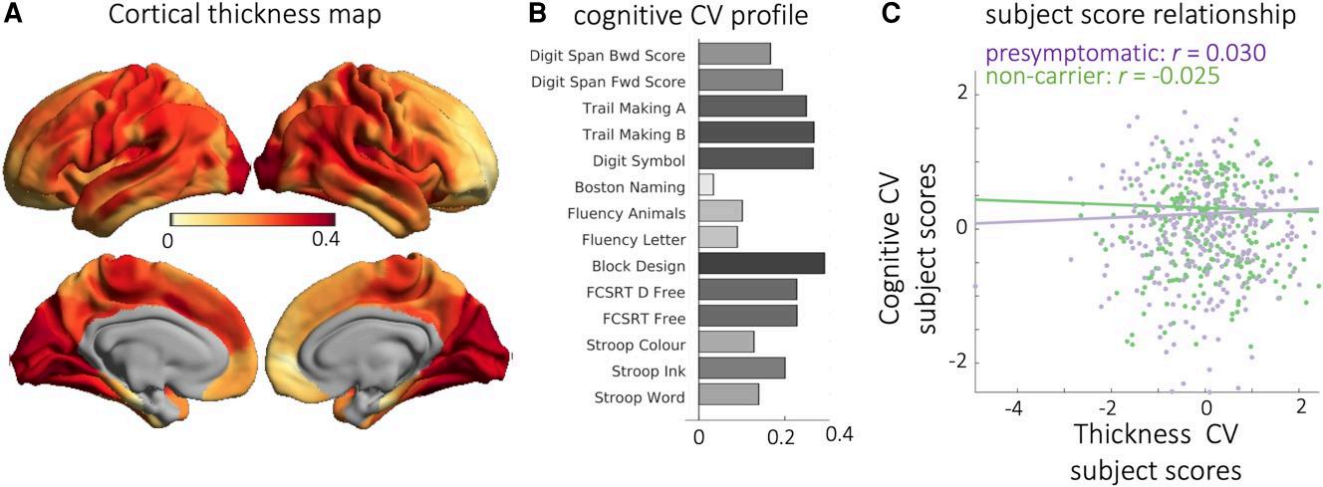
**Link between cortical thickness and cognition using canonical correlation analysis.** (**A**) Spatial distribution of parcellated cortical thickness value (i.e. loading values ranging from −0.085 to 0.42), where light yellow to dark red shading indicates the strength of the positive correlation with the cognitive function profile in **B**. (**C**) Scatter plot represents the relationship (or lack thereof) between cortical thickness and cognition subjects’ scores for presymptomatic carriers (purple) and non-carriers (green) after accounting for age and other covariates. CV = canonical variate.

Canonical correlation analysis of cortical thickness and cognition identified one significant pair of canonical variates (*r* = 0.32, *P* < 0.001). Variable loadings and subject scores reflecting the strong positive relationship between cortical thickness and cognitive performance are shown in Fig. 5. The cortical thickness canonical variate (Thickness-CV) expressed high loadings in frontal (dorsal-lateral prefrontal gyrus, precentral gyrus, paracentral lobule), parietal (postcentral gyrus, superior and inferior parietal lobule), superior temporal gyrus and occipital (lateral and medial occipital cortex) regions (Fig. 5). The Cognition-CV profile expressed positively a large array of cognitive tests, with strongest values on Block design, Trail Making, Digit Symbol, Selective Reminding tests and Digit Span. The positive correlation between cortical surface and cognitive CVs confirms the expected brain-behaviour relationship across the cohort as a whole, between cortical thickness and attention, processing speed, visuospatial ability and working memory.

To understand the structure-cognition relationship in each group and in relation as a function of age, we performed a second-level interaction analysis using a regression model: we entered Cognition-CV subject scores as dependent variable, and Thickness-CV subject scores, genetic status (i.e. carrier of a pathogenic variant or non-carrier), age, and their interactions as independent variables in addition to covariates of no interest (see the ‘Materials and methods’ section). The results indicated that the relationship between cortical thickness and cognition no longer holds after including age and other covariates in the model (Fig. 5). This suggests that the link between cortical thickness and cognition identified by CCA merely reflects age and/or other covariates of no interest in the model. This aligned with research indicating that structural brain-behaviour correlations are often difficult to establish,^108,109^ suggesting that brain structure alone was insufficient to account for the complex relationship between neural processes and observable behaviours.

#### Functional integration supports cognitive performance in presymptomatic gene carriers

**Figure 6:**
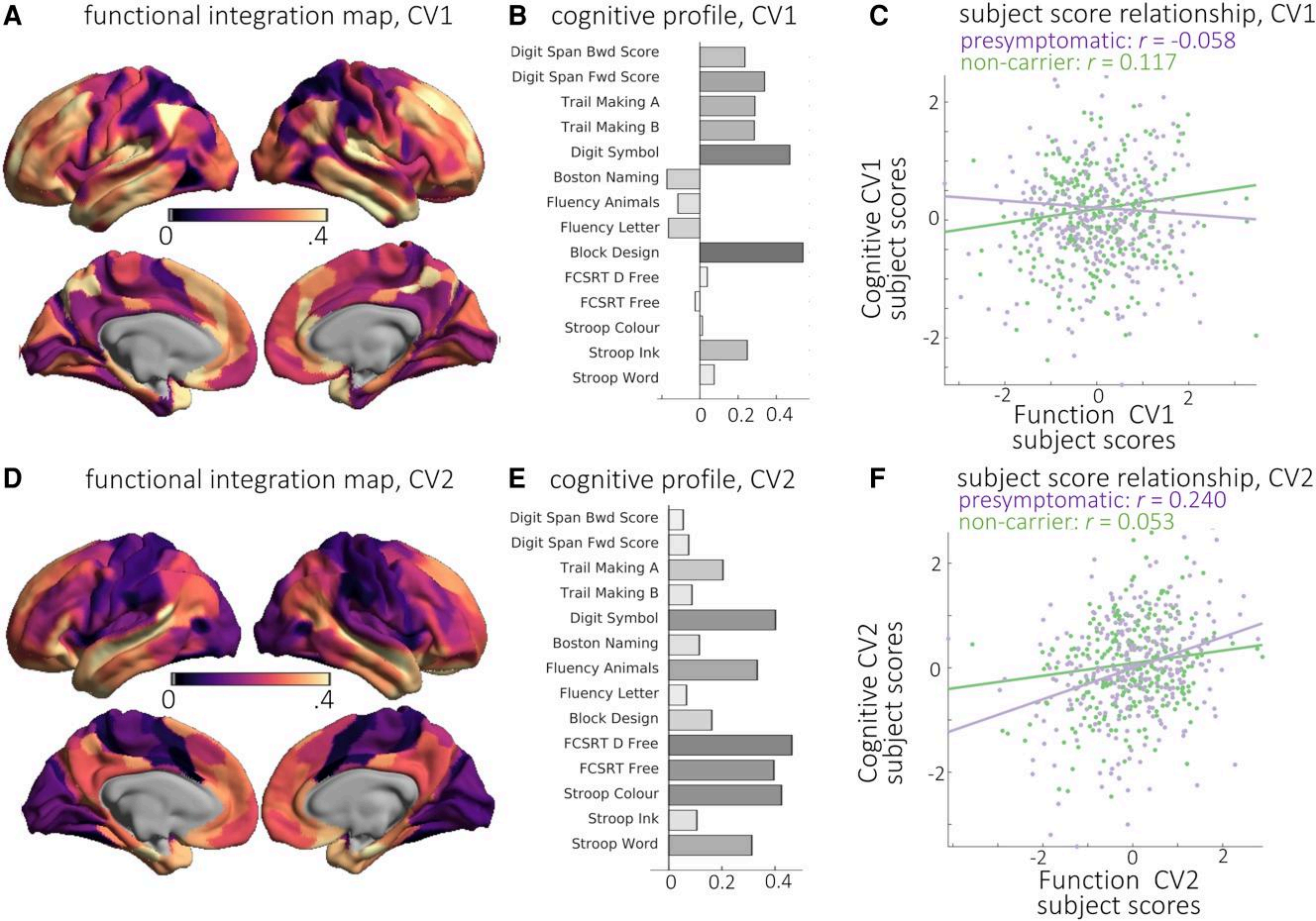
**Link between functional integration and cognition using canonical correlation analysis.** (**A**) Spatial distribution of functional integration nodal values for Canonical Variate 1 (CV1), where dark purple to light yellow colours indicate the strength of the positive correlation with the cognitive function profile in **B**. (**C**) Scatter plot represents the positive relationship between functional integration and cognition subjects’ scores for presymptomatic carriers (purple) and non-carriers (green) after accounting for age and other covariates. (**D**–**F**) Representative results as shown in **A**–**C** for Canonical Variate 2 (CV2).

CCA of functional integration and cognition identified two significant pairs of canonical variates (Function-CV1 and Cognition-CV1, *r* = 0.15, *P* = 0.036; Function-CV2 and Cognition-CV2, *r* = 0.19, *P* = 0.005). Variable loadings and subject scores reflecting the strong positive relationship between functional integration and cognitive performance were shown in Fig. 6A. Function-CV1 expressed high functional integration values in frontal (lateral prefrontal cortex and anterior cingulate), intraparietal regions and inferior temporal regions. Cognition-CV1 expressed positive Digit symbol, Block Design, Digit Span and Trail making tests, alongside negative loadings on Boston Naming and Fluency Tests (Fig. 6B). This first canonical variate pair indicated that higher performance on higher-order cognitive function tests, involving working memory, visuospatial reasoning and processing, and problem-solving was associated with stronger functional integration in the frontoparietal network.

Function-CV2 expressed high functional integration values in frontal (medial frontal cortex, inferior frontal gyrus), parietal (precuneus, intraparietal gyrus) and superior temporal regions (Fig. 6D). Cognition-CV2 expressed positively all tests, with strongest loading values on Digit Symbol, Fluency, Selective Reminding and Stroop tests (Fig. 6E). This second canonical variate pair indicated that higher performance on a wide range of cognitive tests, involving automatic memory retrieval and processing efficiency was associated with stronger functional integration.

To test whether the observed behaviourally-relevant pattern of functional integration was differentially expressed between genetic status groups and age, we constructed a second-level regression model with robust error estimates by including Function-CV subject scores, genetic status, age, their interaction terms as independent variables, and Cognition-CV as dependent variable in addition to covariates of no interest.

We found evidence for significant interaction between genetic status and Function-CV1 (*r* = −0.09, *P* = 0.019) explaining unique variance in Cognition-CV1. We used simple slope analysis and slope difference tests^99-101^ to test formally for differences in the relationship between Function-CV1 and Cognition-CV1 for presymptomatic carriers and non-carriers. The relationship between Function-CV1 and Cognition-CV1 was stronger for non-carriers relative to presymptomatic carriers (*r* = 0.09, *P* = 0.019). This indicated that functional integration in frontoparietal network becomes increasingly important for maintaining higher-order cognitive functions across the health lifespan (Fig. 6C).

For Canonical Variate 2, the interaction between genetic status and Function-CV2 (*r* = 0.10, *P* = 0.019) and Function-CV2 (*r* = 0.15, *P* < 0.001) explained unique variance in Cognition-CV2. Simple slope analysis and slope difference tests revealed that the relationship between Function-CV2 and Cognition-CV2 was stronger for presymptomatic carriers relative to non-carriers (*r* = 0.09, *P* = 0.019). Non-carriers showed no relationship (*r* = 0.053, *P* = 0.191), while carriers showed a highly significant one (*r* = 0.240, *P* < 0.001). These results remained virtually identical when controlling for pathogenic variant type (*C9orf72*, *GRN*, *MAPT*) interaction: *r* = 0.09, *P* = 0.020; presymptomatic carriers: *r* = 0.246, *P* < 0.001; and non-carriers: *r* = 0.059, *P* = 0.151, confirming findings are independent of the specific genetic variant. This indicated that functional integration in medial frontal and temporoparietal regions is increasingly important for presymptomatic carriers to maintain performance (Fig. 6F). This pattern suggested a brain functional resilience mechanism where higher level of functional integration was required to maintain performance when pathology was present.

### Spatial overlap of brain functional resilience maps with brain cell-type distributions

**Figure 7:**
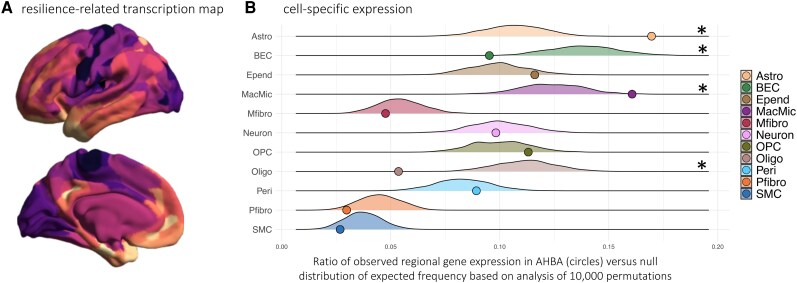
**Spatial correspondence between the functional resilience map expressed by canonical variate 2 and cell-type decomposition.** (**A**) Spatial map of the weighted whole-genome expression profile, correlated with the resilience-induced functional integration map. (**B**) Cell-type decomposition was used to identify cell-type enrichment based on the extent to which the genes are expressed in the transcriptome map in **A**. Gene sets for each cell-type were constructed by thresholding genes with significant loadings. *P*-value < 0.05. Note that results were consistent across a range of thresholds and labelling schemes for cell-type decomposition, see Supplementary Fig. 4. The ratio of genes in each gene set preferentially expressed in eleven distinct cell types (circles) was shown against their null distribution of a model with random selection of all genes (10 000 permutations, *P-value < 0.05). For example, the pericyte ratio was calculated from the number of genes preferentially expressed in pericytes divided by the total number of genes. Cell-type specificity of genes has been described elsewhere.^107^ Astro = astrocytes; BEC = brain endothelial cells; Epend = ependymal; MacMic = macrophage/microglia; Mfibro = meningeal fibroblast; Neuron = neuron; OPC = oligodendrocyte precursor cells; Oligo = oligodendrocytes; Peri = pericytes; Pfibro = perivascular fibroblast; SMC = smooth muscle cells.

We next used normative transcriptomics to identify genes that are normally preferentially expressed in the regions associated with brain functional resilience in presymptomatic FTD (i.e. Function-CV2). Regularized-CCA identified one latent component (*r* = 0.58, *P* = 0.001; Fig. 7). Using cell-type decomposition analysis on cell-type specific gene sets, we determined the ratio of genes in each gene set preferentially expressed across eleven cortical cell types. A complete list of gene-to-cell assignments was available in Yang *et al.*^107^ Gene sets were thresholded to include the genes with significant loadings at *P*-value <0.05 (10 000 permutations) (Fig. 7). The results were consistent across various thresholds and labelling schemes for cell-type decomposition (Supplementary Fig. 4). Highly ranking genes were significantly more expressed in glial cells (astrocytes, macrophage/microglia and oligodendrocytes) and significantly less expressed in brain endothelial cells. Broadly, we found evidence that areas associated with functional resilience in presymptomatic FTD are enriched for expression of genes related to glial cell function and neuroinflammation. These same regions showed under-expression of genes in endothelial cells.

## Discussion

We identified brain-wide structural and functional imaging differences between presymptomatic carriers of FTD-related genetic pathogenic variants and non-carriers from the same families. Despite observing atrophy patterns reflecting disease progression, cognitive performance remained similar between groups. Functional node integration in cognitive control networks was preserved in carriers across age, suggesting a resilience mechanism in those at risk of FTD, before conversion to the symptomatic phase of disease. This resilience was further evidenced in brain-cognition relationships: while grey matter-cognition relationships were consistent across groups, presymptomatic carriers showed stronger associations between functional integration and cognition. The spatial profile of functional resilience corresponded with normative variations in cell-type distributions, indicating that enhanced glial support may underlie this resilience. These findings offer new insights into the resilience mechanisms that support the complex interplay between structural atrophy, functional adaptations, and cognitive preservation in presymptomatic FTD.

People with genetically determined high risk of dementia can maintain good cognitive abilities and successful daily functioning despite significant neuronal loss during presymptomatic stages. The ability to maintain functional integration in the presence of neuropathology suggests a form of resilience,^6,110^ which may play a part in delaying symptoms onset and preserving quality of life in individuals at risk of FTD. The dissociation between structural decline and preserved function can occur to some extent with healthy ageing^2,111^ but is exaggerated in presymptomatic genetic FTD as shown in the current study.

While earlier work on resilience in FTD and related disorders typically focused on global measures or a limited set of brain regions,^4,5,112^ we identified the brain-wide spatial pattern of functional resilience. This comprehensive coverage was an advantage, as neither the functional anatomy of cognition nor the foci of neurodegeneration were confined to singular regions, but rather were distributed across multi-level, interactive networks.^35,36^ To identify the whole brain topology of resilience, we developed a novel multi-dimensional brain network approach.^33^ This delineated the brain-wide pattern of network organization across levels of cortical hierarchy and in relation to cognitive preservation. This methodology offered three key advantages.

First, the brain-wide patterns of functional resilience were used to identify potential mediators and moderators of resilience by linking gene transcription profiles.^47,50^ Our cell-type specific gene expression analysis highlighted the resilience-relevance of genes preferentially expressed in glial cells (astrocytes, macrophage/microglia and oligodendrocytes) and under expressed in endothelial cells.^102,107^ Glial cells are involved in neuronal homeostasis and defence mechanisms against disease conditions, including physiological neuroplasticity, synapse turnover, brain barrier health, and protection against oxidative stress.^113,114^ Specifically, astrocytes enhanced homeostatic buffering through potassium regulation and metabolic coupling, providing lactate support and protein clearance while actively modulating synaptic plasticity.^115^ Microglia showed protective immune responses, clearing early protein clumps and releasing brain-protective factors.^116^ However, regions with high microglial expression may also reflect early neuroinflammation,^117-119^ as microglia can switch from protective to harmful activated states in later disease stages causing functional disruption.^27^ This dual nature suggests beneficial effects in presymptomatic phases may become damaging as FTD progresses. Conversely, the under-expression of endothelial cell genes may reflect reduced vascular inflammation, maintaining blood–brain barrier integrity while shifting toward oligodendrocyte-mediated support rather than endothelial-driven inflammatory responses. This integrated glial response suggests presymptomatic FTD resilience emerges from proactive cellular defence mechanisms that preserve neuronal function through complementary pathways while minimizing potentially harmful vascular activation. Overall, this is well aligned with the idea that glial cells are intricately related to cognitive resilience^120^ and further motivates understanding of the molecular processes underlying defence responses to neurodegeneration and targeted interventions supporting resilience.^121^

Second, the mapping of connectivity estimates to regional effects allowed us to account for atrophy at a regional level (Supplementary Fig. 2). This ensured that the more pronounced functional resilience profile in presymptomatic carriers was independent of regional atrophy, rather than only adjusted for global atrophy.^4,5^ It is worth noting that this framework facilitates the integration of connectomes (e.g. structural and functional connectivity) with regional effects from multiple modalities. While such structure-function integration has been attempted previously using connectivity-based approaches,^2,46^ results in connectivity space are often difficult to interpret. The current approach, mapping connectivity space to brain space, enables more interpretable exploration of structure-function relationships. It also allows integration with grey matter, perfusion, cerebrovascular reactivity, metabolic rates of glucose, oxygen, aerobic glycolysis^122^ and receptor and neurotransmitter maps,^123^ in addition to transcriptomic patterns as demonstrated above. We propose that by adopting this comprehensive, multimodal perspective, one can advance insights and track the complex multilevel changes preceding clinical symptoms in neurodegenerative diseases such as FTD.^112,124-126^ This is especially important where the presymptomatic stages of active disease offer a critical window of opportunity for preventive disease-modifying treatment.^127^

Third, our approach was based on data-driven decomposition that maps functional connectivity patterns to a higher-dimensional space of neuronal effects.^33,35^ This mitigated the bias from vascular contributions to fMRI blood oxygen level-dependent (BOLD) signals,^128,129^ which can be differentially affected by ageing,^130-132^ and systemic-to-vascular interactions.^98,133,134^ By projecting the fMRI BOLD signal onto a set of functional components, we isolated the neuronal components of the signal, while filtering physiological noise from other components.^43^ This was valuable even in the absence of explicit measures of vascular or neuronal signals.^43^

Functional node integration in cognitive control networks (i.e. frontoparietal and default mode networks) was preserved in carriers across age but declined in familial non-carriers. We interpret this in the light of two concurrent processes. First, as shown by Bethlehem *et al.*,^33^ there is an age-related decrease in frontoparietal and default-mode integration. Second, there is a compensatory increase in response to presymptomatic neurodegeneration that maintains function until the capacity for compensatory resilience is overwhelmed.^4,5^ This matches prior reports of widespread connectivity declines, particularly within cognitive control networks.^25,33,34,135-140^ When compared with other approaches—clustering,^136^ within network connectivity^135^ and segregation^137^—this gradient-based integration proved more sensitive to age-related differences.^33^ Importantly, preserved integration correlated with better fluid intelligence performance.^33^ Our findings were consistent, as shown in the Cognition-CV1 analysis, where higher integration in the frontoparietal network was associated with stronger cognitive performance in higher-order cognitive functions, particularly in non-carriers. Similar age-related patterns have also been observed across modalities including magnetoencephalography,^28,29^ cognitive domains,^32,42,44,141,142^ and after accounting for vascular confounds^25,34^ and structural differences.^2,111^ This supports our earlier point that gradients more directly reflect neuronal organization.

In the presymptomatic carriers, by contrast, functional integration remained stable with age, maintaining youth-like or elevated levels. This aligns with literature showing at-risk individuals may temporarily outperform their age-matched non-carriers. For example, APOE ε4 carriers exhibited greater cortical volumes and superior memory performance.^143^ GENFI presymptomatic carriers of pathogenic variants with higher education showed enhanced cognitive performance and slower decline.^144^ Similarly, maintaining connectivity in cognitive control networks facilitated resilience in both preclinical Alzheimer’s disease^145^ and presymptomatic FTD carriers.^5,112^ Interestingly, neurocognitive models^6,146-149^ emphasized compensatory engagement of control networks to sustain performance under neural decline. This suggests recruitment and preservation of cognitive control networks represents a domain-general resilience mechanism across both healthy and pathological ageing, extending these models to presymptomatic neurodegeneration. Importantly, we do not exclude other resilience forms, including domain-specific networks, which could be examined with task-based fMRI to separate domain-general from specialized mechanisms.^42^

Previous studies have reported hyperexcitability and hyperconnectivity in high-dementia-risk individuals.^150-152^ However, without linkage to behaviour, it is unclear whether these reflected compensation or maladaptive dedifferentiation.^42^ By relating functional integration to performance, our findings supported a compensatory interpretation. The canonical variate (Cognition-CV2) reflected preserved automatic retrieval and processing efficiency, linked to medial frontal and temporoparietal integration. This pattern was stronger in presymptomatic FTD, suggesting compensatory up-regulation responding to incipient pathology. While potentially supporting performance during the presymptomatic stages, it could also impose greater metabolic cost^153^ and contribute to subsequent decline, consistent with models of stage-dependent, non-linear network trajectories in neurodegeneration.^4,154^ Differentiating compensation from other mechanisms requires linking reorganization directly to cognitive performance and neuropathology estimates, ideally longitudinally.^11,155^

Despite the large size of the GENFI cohort, this study had several limitations. Genetic groups were not analysed separately due to potential subgroup imbalances and lower statistical power. Similarly, environmental factors and genetic modifiers were not fully addressed.^20,156^ While efforts were made to harmonize data acquisition and reduce site-specific effects, residual scanner variance cannot be ruled out. The cross-sectional design limited causal inferences about the progression of atrophy and resilience effects. Moreover, the study did not differentiate between grey matter atrophy and structural connectivity effects on functional integration, which might be essential as cognition’s dependence on functional integration could be partly conditional on structural integrity, at least in ageing.^2^ While the identified cellular associations were consistent across multiple thresholds and independent cell-type atlases, the study did not involve direct comparisons of gene expression across individuals or disease conditions. Instead, it implied cellular associations through spatial correlations with reference transcriptomic data. Finally, the findings were limited to autosomal dominant FTD and might not generalize to sporadic FTD or might generalize to other forms of dementia. These limitations highlight the need for larger, longitudinal FTD cohorts, with diverse neuroimaging measures,^157^ to better understand gene-specific effects and environmental moderators. They would clarify how network integration preserves cognition across different FTD subtypes and in relation to other dementias. In parallel, studies combining matched ante-mortem functional imaging and post-mortem brain tissue analysis will be critical for validating the cellular mechanisms we propose.

In conclusion, the multivariate data-driven approach revealed that brain functional integration provided resilience, allowing presymptomatic carriers to maintain cognitive performance despite brain atrophy. Localized to cognitive control networks, this resilience was associated with transcriptomic profiles for glial support. This multivariate approach to whole-brain brain function in relation to cognition, genetics and modifiers was well-suited to study the impact of multiple risk factors on neurodegeneration biomarkers before clinical symptoms appear. Our findings have implications for presymptomatic therapy trials, which are likely to rely initially on surrogate markers of brain health rather than clinical end points. Furthermore, our findings open new avenues for therapeutic strategies aimed at enhancing resilience, with targeted interventions for preserving cognitive function in the face of neurodegeneration.

## Supplementary Material

awaf443_Supplementary_Data

## Data Availability

Data were acquired from GENFI data freeze 5. Anonymized data not published within this article will be made available by request from any qualified investigator and can be requested via the GENFI website (https://www.genfi.org/contact-us-2) or via Dementias Platform UK (https://portal.dementiasplatform.uk/Apply). Code and composite data to reproduce manuscript figures and statistical analyses were made available at https://github.com/kamentsvetanov/functional_resilience_ftd. Surface-based analysis of anatomical images were performed in FreeSurfer.^61^ Resting-state fMRI data were pre-processed using FSL pipelines^58,59^ and modules which called relevant functions from SPM12.^60^ Post-processing was based on a GLM-like approach^34^ available at https://github.com/MRC-CBU/riksneurotools/blob/master/GLM/. Visualization of neuro-imaging results was in MRIcroGL and BrainSpace, while ridgeline plots generated using R-based ggridges and ggplot2 (10.32614/CRAN.package.ggridges).^158,159^ Spin permutations used code available at https://github.com/frantisekvasa/rotate_parcellation. Fully-pre-processed transcriptomic data were available at https://figshare.com/articles/dataset/AHBAdata/6852911 and https://github.com/BMHLab/AHBAprocessing. The molecular atlas of the human brain vasculature was made available previously.^107^ Code for cell-type decomposition analysis was available at https://github.com/netneurolab/hansen_genescognition.

## Appendix 1

### The GENFI consortium

Rhian Convery, Martina Bocchetta, David Cash, Sophie Goldsmith, Kiran Samra, David L. Thomas, Thomas Cope, Maura Malpetti, Antonella Alberici, Enrico Premi, Roberto Gasparotti, Emanuele Buratti, Valentina Cantoni, Andrea Arighi, Chiara Fenoglio, Vittoria Borracci, Maria Serpente, Tiziana Carandini, Emanuela Rotondo, Giacomina Rossi, Giorgio Giaccone, Giuseppe Di Fede, Paola Caroppo, Sara Prioni, Veronica Redaelli, David Tang-Wai, Ekaterina Rogaeva, Johanna Krüger, Miguel Castelo-Branco, Morris Freedman, Ron Keren, Sandra Black, Sara Mitchell, Christen Shoesmith, Robart Bartha, Rosa Rademakers, Jackie Poos, Janne M. Papma, Lucia Giannini, Liset de Boer, Julie de Houwer, Rick van Minkelen, Yolande Pijnenburg, Benedetta Nacmias, Camilla Ferrari, Cristina Polito, Gemma Lombardi, Valentina Bessi, Enrico Fainardi, Stefano Chiti, Mattias Nilsson, Henrik Viklund, Melissa Taheri Rydell, Vesna Jelic, Linn Öijerstedt, Tobias Langheinrich, Albert Lladó, Anna Antonell, Jaume Olives, Mircea Balasa, Nuria Bargalló, Sergi Borrego-Ecija, Ana Verdelho, Carolina Maruta, Tiago Costa-Coelho, Gabriel Miltenberger, Frederico Simões do Couto, Alazne Gabilondo, Ioana Croitoru, Mikel Tainta, Myriam Barandiaran, Patricia Alves, Benjamin Bender, David Mengel, Lisa Graf, Annick Vogels, Mathieu Vandenbulcke, Philip Van Damme, Rose Bruffaerts, Koen Poesen, Pedro Rosa-Neto, Maxime Montembault, Raphaella Lara Migliaccio, Ninon Burgos, Daisy Rinaldi, Catharina Prix, Elisabeth Wlasich, Olivia Wagemann, Sonja Schönecker, Alexander Maximilian Bernhardt, Anna Stockbauer, Jolina Lombardi, Sarah Anderl-Straub, Adeline Rollin, Gregory Kuchcinski, Vincent Deramecourt, João Durães, Marisa Lima, Maria João Leitão, Maria Rosario Almeida, Miguel Tábuas-Pereira, Sónia Afonso, João Lemos.
