# Supplementary material for: Cellular signatures underlying functional resilience in presymptomatic frontotemporal dementia
Source: Brain. 2025 Nov 24;149(8):2831–49. doi: 10.1093/brain/awaf443 (PMC13431775; doi:10.1093/brain/awaf443)
Supplement: awaf443_Supplementary_Data [file awaf443_supplementary_data.zip › brain-2025-00549-File011.pdf]

The GENFI Consortium Author List:

Author	Affiliation 1
Rhian Convery	Department of Neurodegenerative Disease, Dementia Research Centre, UCL Queen Square Institute of Neurology, London, UK
Martina Bocchetta	Department of Neurodegenerative Disease, Dementia Research Centre, UCL Queen Square Institute of Neurology, London, UK
David Cash	Department of Neurodegenerative Disease, Dementia Research Centre, UCL Queen Square Institute of Neurology, London, UK
Sophie Goldsmith	Department of Neurodegenerative Disease, Dementia Research Centre, UCL Queen Square Institute of Neurology, London, UK
Kiran Samra	Department of Neurodegenerative Disease, Dementia Research Centre, UCL Queen Square Institute of Neurology, London, UK
David L. Thomas	Neuroimaging Analysis Centre, Department of Brain Repair and Rehabilitation, UCL Institute of Neurology, Queen Square, London, UK
Thomas Cope	Cambridge University Hospitals NHS Trust, Cambridge UK
Maura Malpetti	Department of Clinical Neurosciences, University of Cambridge, Cambridge, UK
Antonella Alberici	Centre for Neurodegenerative Disorders, Department of Clinical and Experimental Sciences, University of Brescia, Brescia, Italy
Enrico Premi	Stroke Unit, ASST Brescia Hospital, Brescia, Italy
Roberto Gasparotti	Neuroradiology Unit, University of Brescia, Brescia, Italy
Emanuele Buratti	ICGEB Trieste, Italy
Valentina Cantoni	Centre for Neurodegenerative Disorders, Department of Clinical and Experimental Sciences, University of Brescia, Brescia, Italy
Andrea Arighi	Fondazione IRCCS Ca’ Granda Ospedale Maggiore Policlinico, Neurodegenerative Diseases Unit, Milan, Italy
Chiara Fenoglio	University of Milan, Centro Dino Ferrari, Milan, Italy
Vittoria Borracci	Fondazione IRCCS Ca’ Granda Ospedale Maggiore Policlinico, Neurodegenerative Diseases Unit, Milan, Italy
Maria Serpente	Fondazione IRCCS Ca’ Granda Ospedale Maggiore Policlinico, Neurodegenerative Diseases Unit, Milan, Italy
Tiziana Carandini	Fondazione IRCCS Ca’ Granda Ospedale Maggiore Policlinico, Neurodegenerative Diseases Unit, Milan, Italy
Emanuela Rotondo	Fondazione IRCCS Ca’ Granda Ospedale Maggiore Policlinico, Neurodegenerative Diseases Unit, Milan, Italy
Giacomina Rossi	Fondazione IRCCS Istituto Neurologico Carlo Besta, Milano, Italy

Giorgio Giaccone	Fondazione IRCCS Istituto Neurologico Carlo Besta, Milano, Italy
Giuseppe Di Fede	Fondazione IRCCS Istituto Neurologico Carlo Besta, Milano, Italy
Paola Caroppo	Fondazione IRCCS Istituto Neurologico Carlo Besta, Milano, Italy
Sara Prioni	Fondazione IRCCS Istituto Neurologico Carlo Besta, Milano, Italy
Veronica Redaelli	Fondazione IRCCS Istituto Neurologico Carlo Besta, Milano, Italy
David Tang-Wai	The University Health Network, Krembil Research Institute, Toronto, Canada
Ekaterina Rogaeva	Tanz Centre for Research in Neurodegenerative Diseases, University of Toronto, Toronto, Canada
Johanna Krüger	Research Unit of Clinical Medicine, Neurology, University of Oulu, Oulu, Finland
Miguel Castelo-Branco	Faculty of Medicine, ICNAS, CIBIT, University of Coimbra, Coimbra, Portugal.
Morris Freedman	Baycrest Health Sciences, Rotman Research Institute, University of Toronto, Toronto, Canada
Ron Keren	The University Health Network, Toronto Rehabilitation Institute, Toronto, Canada
Sandra Black	Sunnybrook Health Sciences Centre, Sunnybrook Research Institute, University of Toronto, Toronto, Canada
Sara Mitchell	Sunnybrook Health Sciences Centre, Sunnybrook Research Institute, University of Toronto, Toronto, Canada
Christen Shoesmith	Department of Clinical Neurological Sciences, University of Western Ontario, London, Ontario, Canada
Robart Bartha	Department of Medical Biophysics, The University of Western Ontario, London, Ontario, Canada
Rosa Rademakers	Center for Molecular Neurology, University of Antwerp
Jackie Poos	Department of Neurology, Erasmus Medical Center, Rotterdam, Netherlands
Janne M. Papma	Department of Neurology, Erasmus Medical Center, Rotterdam, Netherlands
Lucia Giannini	Department of Neurology, Erasmus Medical Center, Rotterdam, Netherlands
Liset de Boer	Department of Neurology, Erasmus Medical Center, Rotterdam, Netherlands
Julie de Houwer	Department of Neurology, Erasmus Medical Center, Rotterdam, Netherlands
Rick van Minkelen	Department of Clinical Genetics, Erasmus Medical Center, Rotterdam, Netherlands
Yolande Pijnenburg	Amsterdam University Medical Centre, Amsterdam VUMC, Amsterdam, Netherlands
Benedetta Nacmias	Department of Neuroscience, Psychology, Drug Research and Child Health, University of Florence, Florence, Italy
Camilla Ferrari	Department of Neuroscience, Psychology, Drug Research and Child Health, University of Florence, Florence, Italy

Cristina Polito	Department of Biomedical, Experimental and Clinical Sciences “Mario Serio”, Nuclear Medicine Unit, University of Florence, Florence, Italy
Gemma Lombardi	Department of Neuroscience, Psychology, Drug Research and Child Health, University of Florence, Florence, Italy
Valentina Bessi	Department of Neuroscience, Psychology, Drug Research and Child Health, University of Florence, Florence, Italy
Enrico Fainardi	Neuroradiology Unit, Department of Experimental and Clinical Biomedical Sciences, University of Florence, Florence, Italy
Stefano Chiti	Neuroradiology Unit, Department of Experimental and Clinical Biomedical Sciences, University of Florence, Florence, Italy.
Mattias Nilsson	Department of Clinical Neuroscience, Karolinska Institutet, Stockholm, Sweden
Henrik Viklund	Karolinska University Hospital Huddinge
Melissa Taheri Rydell	Department of Neurobiology, Care Sciences and Society; Center for Alzheimer Research, Division of Neurogeriatrics, , Bioclinicum, Karolinska Institutet, Solna, Sweden
Vesna Jelic	Department of Neurobiology, Care Sciences and Society; Division of Clinical Geriatrics, Karolinska Institutet, Stockholm, Sweden
Linn Öijerstedt	Department of Neurobiology, Care Sciences and Society; Center for Alzheimer Research, Division of Neurogeriatrics, , Bioclinicum, Karolinska Institutet, Solna, Sweden
Tobias Langheinrich	Division of Neuroscience and Experimental Psychology, Wolfson Molecular Imaging Centre, University of Manchester, Manchester, UK
Albert Lladó	Alzheimer’s disease and Other Cognitive Disorders Unit, Neurology Service, Hospital Clínic, Barcelona, Spain
Anna Antonell	Alzheimer’s disease and Other Cognitive Disorders Unit, Neurology Service, Hospital Clínic, Barcelona, Spain
Jaume Olives	Alzheimer’s disease and Other Cognitive Disorders Unit, Neurology Service, Hospital Clínic, Barcelona, Spain
Mircea Balasa	Alzheimer’s disease and Other Cognitive Disorders Unit, Neurology Service, Hospital Clínic, Barcelona, Spain
Nuria Bargalló	Imaging Diagnostic Center, Hospital Clínic, Barcelona, Spain
Sergi Borrego-Ecija	Alzheimer’s disease and Other Cognitive Disorders Unit, Neurology Service, Hospital Clínic, Barcelona, Spain
Ana Verdelho	Department of Neurosciences and Mental Health, Centro Hospitalar Lisboa Norte - Hospital de Santa Maria & Faculty of Medicine, University of Lisbon, Lisbon, Portugal
Carolina Maruta	Laboratory of Language Research, Centro de Estudos Egas Moniz, Faculty of Medicine, University of Lisbon, Lisbon, Portugal
Tiago Costa-Coelho	Faculty of Medicine, University of Lisbon, Lisbon, Portugal

Gabriel Miltenberger	Faculty of Medicine, University of Lisbon, Lisbon, Portugal
Frederico Simões do Couto	Faculdade de Medicina, Universidade Católica Portuguesa
Alazne Gabilondo	Cognitive Disorders Unit, Department of Neurology, Donostia University Hospital, San Sebastian, Gipuzkoa, Spain
Ioana Croitoru	Instituto de Investigación Sanitaria Biogipuzkoa, Neurosciences Area, Group of Neurodegenerative Diseases, San Sebastian, Spain.
Mikel Tainta	Instituto de Investigación Sanitaria Biogipuzkoa, Neurosciences Area, Group of Neurodegenerative Diseases, San Sebastian, Spain.
Myriam Barandiaran	Cognitive Disorders Unit, Department of Neurology, Donostia University Hospital, San Sebastian, Gipuzkoa, Spain
Patricia Alves	Instituto de Investigación Sanitaria Biogipuzkoa, Neurosciences Area, Group of Neurodegenerative Diseases, San Sebastian, Spain.
Benjamin Bender	Department of Diagnostic and Interventional Neuroradiology, University of Tübingen, Tübingen, Germany
David Mengel	Department of Neurodegenerative Diseases, Hertie-Institute for Clinical Brain Research and Center of Neurology, University of Tübingen, Tübingen, Germany
Lisa Graf	Department of Neurodegenerative Diseases, Hertie-Institute for Clinical Brain Research and Center of Neurology, University of Tübingen, Tübingen, Germany
Annick Vogels	Department of Human Genetics, KU Leuven, Leuven, Belgium
Mathieu Vandenbulcke	Geriatric Psychiatry Service, University Hospitals Leuven, Belgium; Neuropsychiatry, Department of Neurosciences, KU Leuven, Leuven, Belgium
Philip Van Damme	Neurology Service, University Hospitals Leuven, Belgium; Laboratory for Neurobiology, VIB-KU Leuven Centre for Brain Research, Leuven, Belgium
Rose Bruffaerts	Department of Biomedical Sciences, University of Antwerp, Antwerp, Belgium; Biomedical Research Institute, Hasselt University, 3500 Hasselt, Belgium
Koen Poesen	Laboratory for Molecular Neurobiomarker Research, KU Leuven, Leuven, Belgium
Pedro Rosa-Neto	Translational Neuroimaging Laboratory, McGill Centre for Studies in Aging, McGill University, Montreal, Québec, Canada
Maxime Montembault	Douglas Research Centre, Department of Psychiatry, McGill University, Montreal, Québec, Canada
Raphaella Lara Migliaccio	Sorbonne Université, Paris Brain Institute – Institut du Cerveau – ICM, Inserm U1127, CNRS UMR 7225, AP-HP - Hôpital Pitié-Salpêtrière, Paris, France
Ninon Burgos	Sorbonne Université, Paris Brain Institute – Institut du Cerveau – ICM, Inserm U1127, CNRS UMR 7225, AP-HP - Hôpital Pitié-Salpêtrière, Paris, France
Daisy Rinaldi	Sorbonne Université, Paris Brain Institute – Institut du Cerveau – ICM, Inserm U1127,

	CNRS UMR 7225, AP-HP - Hôpital Pitié-Salpêtrière, Paris, France
Catharina Prix	Neurologische Klinik, Ludwig-Maximilians-Universität München, Munich, Germany
Elisabeth Wlasich	Neurologische Klinik, Ludwig-Maximilians-Universität München, Munich, Germany
Olivia Wagemann	Neurologische Klinik, Ludwig-Maximilians-Universität München, Munich, Germany
Sonja Schönecker	Neurologische Klinik, Ludwig-Maximilians-Universität München, Munich, Germany
Alexander Maximilian Bernhardt	Neurologische Klinik, Ludwig-Maximilians-Universität München, Munich, Germany
Anna Stockbauer	Neurologische Klinik, Ludwig-Maximilians-Universität München, Munich, Germany
Jolina Lombardi	Department of Neurology, University of Ulm, Ulm
Sarah Anderl-Straub	Department of Neurology, University of Ulm, Ulm, Germany
Adeline Rollin	CHU, CNR-MAJ, Labex Distalz, LiCEND Lille, France
Gregory Kuchcinski	Univ Lille, France; Inserm 1172, Lille, France; CHU, CNR-MAJ, Labex Distalz, LiCEND Lille, France
Vincent Deramecourt	Univ Lille, France; Inserm 1172, Lille, France; CHU, CNR-MAJ, Labex Distalz, LiCEND Lille, France
João Durães	Neurology Department, Centro Hospitalar e Universitario de Coimbra, Coimbra, Portugal
Marisa Lima	Neurology Department, Centro Hospitalar e Universitario de Coimbra, Coimbra, Portugal
Maria João Leitão	Centre of Neurosciences and Cell Biology, Universidade de Coimbra, Coimbra, Portugal
Maria Rosario Almeida	Faculty of Medicine, University of Coimbra, Coimbra, Portugal
Miguel Tábuas-Pereira	Neurology Department, Centro Hospitalar e Universitario de Coimbra, Coimbra, Portugal; Faculty of Medicine, University of Coimbra, Coimbra, Portugal.
Sónia Afonso	Instituto Ciencias Nucleares Aplicadas a Saude, Universidade de Coimbra, Coimbra, Portugal
João Lemos	Faculty of Medicine, University of Coimbra, Coimbra, Portugal
